# Bis(5-amino-3-carb­oxy-1*H*-1,2,4-triazol-4-ium) dihydrogenphosphate nitrate 5-amino-1*H*-1,2,4-triazol-4-ium-3-carboxyl­ate

**DOI:** 10.1107/S1600536812014481

**Published:** 2012-04-06

**Authors:** Fadila Berrah, Rafika Bouchene, Sofiane Bouacida, Jean-Claude Daran

**Affiliations:** a Laboratoire de Chimie Appliquée et Technologie des Matériaux, LCATM, Université Larbi Ben M’Hidi, 04000 Oum El Bouaghi, Algeria; b Unité de Recherche de Chimie de l’Environnement et Moléculaire Structurale, CHEMS, Faculté des Sciences Exactes, Université Mentouri Constantine 25000, Algeria; c Laboratoire de Chimie de Coodination, UPR–CNRS 8241, 205, Route de Narbonne, 31077 Toulouse cedex 04, France

## Abstract

In the title compound, 2C_3_H_5_N_4_O_2_^+^·H_2_PO_4_^−^·NO_3_^−^·C_3_H_4_N_4_O_2_, three independent 5-amino-1*H*-1,2,4-triazol-3-carb­oxy­lic acid moieties are observed. Two are in the form of cations, while the third is in the zwitterionic form. The triazole rings in the two cations are almost coplanar, making an angle of 4.11 (7)°. Layers parallel to the (20-1) plane, resulting from hydrogen bonding of the organic mol­ecules and the nitrate anions, are linked *via* H_2_PO_4_^−^ infinite zigzag chains running parallel to the *c* axis. The crystal studied was an inversion twin, with refined components of 0.33 (7) and 0.67 (7).

## Related literature

For structural studies of related compounds, see: Berrah *et al.* (2011 ▶, 2012 ▶); Fernandes *et al.* (2011 ▶); Ouakkaf *et al.* (2011 ▶). For hydrogen-bond motifs, see: Etter *et al.* (1990 ▶); Grell *et al.* (1999 ▶).
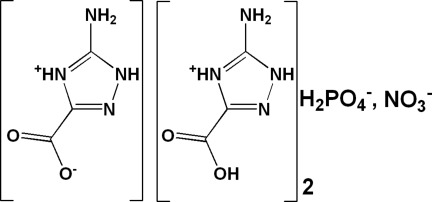


## Experimental

### 

#### Crystal data

2C_3_H_5_N_4_O_2_^+^·NO_3_^−^·H_2_PO_4_^−^·C_3_H_4_N_4_O_2_
*M* *_r_* = 545.32
Monoclinic, 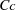

*a* = 19.2249 (13) Å
*b* = 13.2036 (7) Å
*c* = 7.7468 (5) Å
β = 101.079 (7)°
*V* = 1929.8 (2) Å^3^
*Z* = 4
Mo *K*α radiation
μ = 0.25 mm^−1^
*T* = 180 K
0.45 × 0.43 × 0.16 mm

#### Data collection

Agilent Xcalibur Sapphire1 long-nozzle diffractometer
Absorption correction: multi-scan (*CrysAlis PRO*; Agilent, 2011 ▶) *T*_min_ = 0.832, *T*_max_ = 1.000
10050 measured reflections
3836 independent reflections
3735 reflections with *I* > 2σ(*I*)
*R*_int_ = 0.023

#### Refinement

*R*[*F* ^2^ > 2σ(*F* ^2^)] = 0.027
*wR*(*F* ^2^) = 0.067
*S* = 1.05
3836 reflections
330 parameters
2 restraints
H-atom parameters constrained
Δρ_max_ = 0.23 e Å^−3^
Δρ_min_ = −0.27 e Å^−3^
Absolute structure: Flack (1983 ▶), 1858 Friedel pairs
Flack parameter: 0.33 (7)

### 

Data collection: *CrysAlis PRO* (Agilent, 2011 ▶); cell refinement: *CrysAlis PRO*; data reduction: *CrysAlis PRO*; program(s) used to solve structure: *SIR2002* (Burla *et al.*, 2005 ▶); program(s) used to refine structure: *SHELXL97* (Sheldrick, 2008 ▶); molecular graphics: *ORTEPIII* (Burnett & Johnson, 1996 ▶), *ORTEP-3 for Windows* (Farrugia, 1997 ▶) and *DIAMOND* (Brandenburg & Berndt, 2001 ▶); software used to prepare material for publication: *WinGX* (Farrugia, 1999 ▶).

## Supplementary Material

Crystal structure: contains datablock(s) global, I. DOI: 10.1107/S1600536812014481/fj2538sup1.cif

Structure factors: contains datablock(s) I. DOI: 10.1107/S1600536812014481/fj2538Isup2.hkl

Supplementary material file. DOI: 10.1107/S1600536812014481/fj2538Isup3.cml

Additional supplementary materials:  crystallographic information; 3D view; checkCIF report

## Figures and Tables

**Table 1:** Hydrogen-bond geometry (Å, °)

*D*—H⋯*A*	*D*—H	H⋯*A*	*D*⋯*A*	*D*—H⋯*A*
N1*B*—H3*B*⋯O7	0.86	2.24	3.030 (2)	152
N4*C*—H4*C*⋯O5	0.86	1.92	2.765 (2)	169
N4*B*—H4*B*⋯O6	0.86	1.92	2.780 (2)	177
O2—H2⋯O1*C*^i^	0.82	1.77	2.5563 (19)	160
N4*A*—H4*A*⋯O7	0.86	1.93	2.784 (2)	172
O1*B*—H1*B*⋯O3	0.82	1.65	2.4648 (19)	175
O4—H4⋯O3^ii^	0.82	1.92	2.671 (2)	151
O1*A*—H1*A*⋯O1^iii^	0.82	1.62	2.423 (2)	166

Symmetry codes: (i) 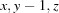; (ii) 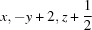; (iii) 
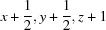
.

**Fig. 1.**
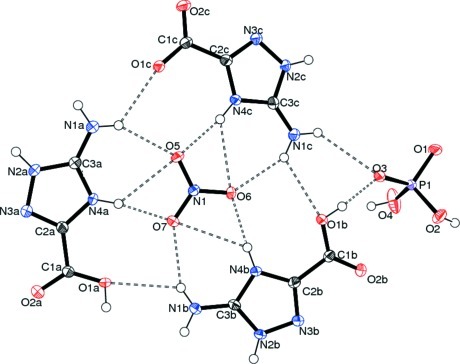
The asymmetric unit of (I) with the atom-labelling scheme. Displacement ellipsoids are drawn at the 50% probability level. H atoms are represented as small spheres of arbitrary radii. Hydrogen bonds are shown as dashed lines.

**Fig. 2.**
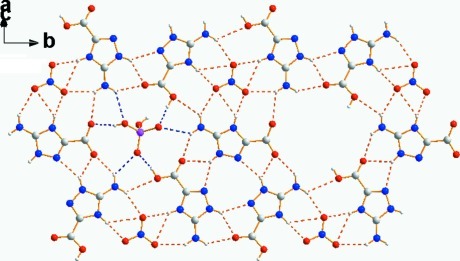
Partial packing view showing layers parallel to (20–1) plane and R66 (18) rings. Only one H2PO4- is represented to show how it fills the rings. Hydrogen bonds are shown as dashed lines.

**Fig. 3.**
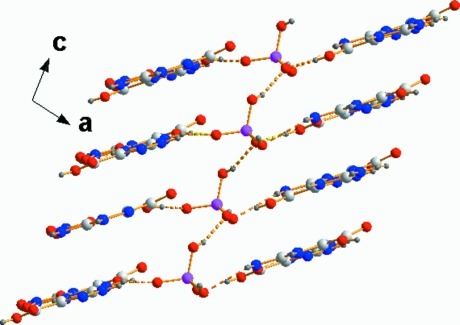
Partial packing view showing H2PO4- infinite zigzag chain running parallel to [001]direction and how it links the layers together. Hydrogen bonds are shown as dashed lines.

## supplementary crystallographic information

### Comment

Synthesis we have undertaken using 1,2,4-triazol derivatives and various inorganic acids (nitric, sulfuric, phosphoric acids and their mixtures) have permitted obtaining hybrids involving sulfate and nitrate anions (Berrah *et al.*, 2012; Ouakkaf *et al.*, 2011) and the title compound which involves a mixture of dihydrgenphosphate and nitrate anions. The comparison between networks observed in these structures make clear the influence of the anion upon the hydrogen bonds patterns encountered.

The asymmetric unit in this compound consists of two cations (A and B), one zwitterium (C), one dihydrogenphosphate anion and one nitrate anion (Fig.1). Bond distances and angles observed in the different entities, present no unusual features and are consistent with those reported previously (Berrah *et al.*, 2011, 2012; Fernandes *et al.*, 2011; Ouakkaf *et al.*, 2011). The triazol rings in (A) and (B) are almost coplanar making an angle of 4.11 (7)°; while they form with the ring in (C) dihedral angles of 8.64 (5)° and 9.62 (6)° respectively.

The title compound shows a three-dimensional packing where organic molecules and nitrate anions, linked by means of O—H···O and N—H···O contacts, lie in layers stacked parallel to (20–1) plane and in which *R*^6^_6_ (18) rings (Etter *et al.*, 1990; Grell *et al.*, 1999) are observed (Fig. 2) (Table 1). H_2_PO_4_^-^ anions form infinite zigzag chains running parallel to the *c* axis; which pass through the *R*^6^_6_ (18) rings to connect the layers together (Fig. 3).

### Experimental

Colourless crystals of compound (I) were obtained by the slow evaporation of a water-methanol (1:1)solution of 5-amino-1,2,4 triazol-1*H*- 3-carboxylic acid hydrate and a mixture of nitric and phosphoric acids in a 1:1 stoichiometric ratio.

### Refinement

All H atoms attached to N atoms and O atom were fixed geometrically and treated as riding with N—H = 0.86 Å and O—H = 0.82 Å with *U*_iso_(H) = 1.2*U*_eq_(N) or *U*_iso_(H) = 1.5*U*_eq_(O).

The value of the Flack parameter, 0.33 (7), suggests the occurrence of a twin by inversion.

### Crystal data

**Table:**

2C_3_H_5_N_4_O_2_^+^·NO^3^^−^·H_2_PO_4_^−^·C_3_H_4_N_4_O_2_	*F*(000) = 1120
*M**_r_* = 545.32	*D*_x_ = 1.877 Mg m^−^^3^
Monoclinic, *C**c*	Mo *K*α radiation, λ = 0.71073 Å
*a* = 19.2249 (13) Å	Cell parameters from 8428 reflections
*b* = 13.2036 (7) Å	θ = 3.1–28.3°
*c* = 7.7468 (5) Å	µ = 0.25 mm^−^^1^
β = 101.079 (7)°	*T* = 180 K
*V* = 1929.8 (2) Å^3^	Box, colourless
*Z* = 4	0.45 × 0.43 × 0.16 mm

### Data collection

**Table:**

Agilent Xcalibur Sapphire1 long-nozzle diffractometer	3836 independent reflections
Radiation source: fine-focus sealed tube	3735 reflections with *I* > 2σ(*I*)
Graphite monochromator	*R*_int_ = 0.023
Detector resolution: 8.2632 pixels mm^-1^	θ_max_ = 26.4°, θ_min_ = 3.1°
ω scans	*h* = −24→23
Absorption correction: multi-scan (*CrysAlis PRO*; Agilent, 2011)	*k* = −16→16
*T*_min_ = 0.832, *T*_max_ = 1.000	*l* = −9→9
10050 measured reflections	

### Refinement

**Table:**

Refinement on *F*^2^	Secondary atom site location: difference Fourier map
Least-squares matrix: full	Hydrogen site location: inferred from neighbouring sites
*R*[*F*^2^ > 2σ(*F*^2^)] = 0.027	H-atom parameters constrained
*wR*(*F*^2^) = 0.067	*w* = 1/[σ^2^(*F*_o_^2^) + (0.0378*P*)^2^ + 0.9492*P*] where *P* = (*F*_o_^2^ + 2*F*_c_^2^)/3
*S* = 1.05	(Δ/σ)_max_ = 0.008
3836 reflections	Δρ_max_ = 0.23 e Å^−^^3^
330 parameters	Δρ_min_ = −0.27 e Å^−^^3^
2 restraints	Absolute structure: Flack (1983), 1858 Friedel pairs
Primary atom site location: structure-invariant direct methods	Flack parameter: 0.33 (7)

### Special details

**Table:**

Experimental. Absorption correction: empirical absorption correction using spherical harmonics, implemented in SCALE3 ABSPACK scaling algorithm. CrysAlisPro (Agilent Technologies, 2011)
Geometry. All e.s.d.'s (except the e.s.d. in the dihedral angle between two l.s. planes) are estimated using the full covariance matrix. The cell e.s.d.'s are taken into account individually in the estimation of e.s.d.'s in distances, angles and torsion angles; correlations between e.s.d.'s in cell parameters are only used when they are defined by crystal symmetry. An approximate (isotropic) treatment of cell e.s.d.'s is used for estimating e.s.d.'s involving l.s. planes.
Refinement. Refinement of *F*^2^ against ALL reflections. The weighted *R*-factor *wR* and goodness of fit *S* are based on *F*^2^, conventional *R*-factors *R* are based on *F*, with *F* set to zero for negative *F*^2^. The threshold expression of *F*^2^ > σ(*F*^2^) is used only for calculating *R*-factors(gt) *etc*. and is not relevant to the choice of reflections for refinement. *R*-factors based on *F*^2^ are statistically about twice as large as those based on *F*, and *R*- factors based on ALL data will be even larger.

### Fractional atomic coordinates and isotropic or equivalent isotropic displacement parameters (Å^2^)

**Table:**

	*x*	*y*	*z*	*U*_iso_*/*U*_eq_	
O6	0.41566 (8)	1.31611 (10)	0.5894 (2)	0.0222 (3)	
N3C	0.18588 (9)	1.43700 (13)	0.1472 (2)	0.0236 (4)	
O1C	0.30311 (8)	1.62763 (11)	0.3449 (2)	0.0272 (3)	
N1B	0.58554 (9)	1.25858 (12)	0.9762 (2)	0.0217 (4)	
H5B	0.6216	1.2555	1.061	0.026*	
H3B	0.5677	1.3164	0.9405	0.026*	
N4C	0.28426 (9)	1.41292 (12)	0.3399 (2)	0.0201 (4)	
H4C	0.3233	1.4265	0.4111	0.024*	
N1	0.43355 (9)	1.40196 (12)	0.6489 (2)	0.0184 (3)	
C3C	0.25660 (10)	1.32115 (15)	0.3022 (3)	0.0187 (4)	
O2C	0.19992 (9)	1.64211 (12)	0.1575 (2)	0.0349 (4)	
N2C	0.19719 (9)	1.33628 (12)	0.1833 (2)	0.0219 (4)	
H2C	0.1695	1.2886	0.1356	0.026*	
O7	0.48583 (8)	1.41235 (11)	0.7696 (2)	0.0276 (3)	
C1C	0.24818 (11)	1.59375 (15)	0.2508 (3)	0.0215 (4)	
O5	0.39976 (9)	1.47811 (11)	0.5845 (2)	0.0313 (4)	
N1C	0.28090 (10)	1.23307 (13)	0.3647 (2)	0.0266 (4)	
H5C	0.3198	1.2297	0.4408	0.032*	
H3C	0.2579	1.1786	0.3296	0.032*	
C2C	0.23928 (11)	1.48136 (15)	0.2447 (3)	0.0202 (4)	
P1	0.27249 (3)	0.89899 (3)	0.30351 (6)	0.01592 (11)	
O2B	0.43278 (7)	0.92432 (10)	0.59606 (18)	0.0210 (3)	
N2B	0.57956 (9)	1.08016 (13)	0.9423 (2)	0.0198 (3)	
H2B	0.6152	1.0654	1.0238	0.024*	
N4B	0.50067 (9)	1.16518 (12)	0.7706 (2)	0.0167 (3)	
H4B	0.475	1.2135	0.7182	0.02*	
N3B	0.53912 (9)	1.01015 (12)	0.8405 (2)	0.0194 (3)	
C2B	0.49164 (10)	1.06425 (14)	0.7377 (2)	0.0167 (4)	
C3B	0.55750 (10)	1.17458 (15)	0.9007 (2)	0.0159 (4)	
O3	0.30722 (7)	0.99921 (10)	0.27601 (17)	0.0198 (3)	
C3A	0.47957 (10)	1.70083 (15)	0.7432 (3)	0.0186 (4)	
O2	0.32459 (7)	0.81370 (10)	0.2805 (2)	0.0247 (3)	
H2	0.309	0.7593	0.3072	0.037*	
O1	0.20396 (9)	0.88190 (12)	0.1753 (3)	0.0359 (4)	
N3A	0.56813 (9)	1.75022 (12)	0.9580 (2)	0.0189 (3)	
N2A	0.51436 (9)	1.78030 (13)	0.8257 (2)	0.0202 (3)	
H2A	0.5042	1.8425	0.7988	0.024*	
O2A	0.65270 (7)	1.62929 (11)	1.20239 (19)	0.0233 (3)	
N1A	0.42476 (10)	1.70396 (14)	0.6126 (2)	0.0266 (4)	
H5A	0.4079	1.7614	0.572	0.032*	
H3A	0.4058	1.6486	0.5677	0.032*	
C1B	0.43737 (10)	1.01611 (14)	0.6006 (3)	0.0164 (4)	
N4A	0.51196 (9)	1.61845 (12)	0.8229 (2)	0.0181 (3)	
H4A	0.5014	1.5563	0.7966	0.022*	
O1B	0.40060 (7)	1.08030 (10)	0.49496 (18)	0.0185 (3)	
H1B	0.3709	1.05	0.4234	0.028*	
O4	0.25679 (10)	0.89062 (14)	0.4902 (2)	0.0431 (5)	
H4	0.2766	0.9369	0.551	0.065*	
O1A	0.60814 (8)	1.49336 (11)	1.0444 (2)	0.0267 (3)	
H1A	0.6426	1.4631	1.0994	0.04*	
C1A	0.61377 (10)	1.58682 (15)	1.0807 (3)	0.0180 (4)	
C2A	0.56512 (10)	1.65246 (15)	0.9543 (3)	0.0174 (4)	

### Atomic displacement parameters (Å^2^)

**Table:**

	*U*^11^	*U*^22^	*U*^33^	*U*^12^	*U*^13^	*U*^23^
O6	0.0208 (7)	0.0113 (6)	0.0303 (7)	−0.0030 (5)	−0.0055 (6)	−0.0021 (6)
N3C	0.0210 (9)	0.0151 (8)	0.0305 (10)	−0.0020 (7)	−0.0057 (8)	0.0017 (7)
O1C	0.0224 (8)	0.0126 (7)	0.0398 (9)	−0.0015 (6)	−0.0109 (7)	0.0016 (6)
N1B	0.0206 (9)	0.0172 (8)	0.0232 (8)	−0.0014 (7)	−0.0061 (7)	0.0008 (7)
N4C	0.0175 (9)	0.0147 (8)	0.0243 (9)	−0.0010 (6)	−0.0059 (7)	0.0012 (6)
N1	0.0166 (8)	0.0133 (8)	0.0235 (8)	0.0012 (6)	−0.0006 (7)	0.0016 (6)
C3C	0.0191 (10)	0.0172 (9)	0.0193 (9)	−0.0020 (7)	0.0020 (8)	−0.0008 (7)
O2C	0.0277 (8)	0.0176 (8)	0.0494 (10)	0.0007 (7)	−0.0172 (7)	0.0056 (7)
N2C	0.0192 (9)	0.0122 (8)	0.0302 (9)	−0.0038 (6)	−0.0056 (7)	−0.0013 (7)
O7	0.0244 (8)	0.0181 (7)	0.0326 (8)	0.0005 (6)	−0.0138 (7)	−0.0017 (6)
C1C	0.0196 (10)	0.0153 (10)	0.0273 (11)	0.0014 (8)	−0.0012 (8)	0.0028 (8)
O5	0.0286 (8)	0.0129 (7)	0.0439 (9)	0.0020 (6)	−0.0145 (7)	0.0045 (6)
N1C	0.0271 (10)	0.0136 (8)	0.0333 (10)	−0.0019 (7)	−0.0091 (8)	0.0001 (7)
C2C	0.0171 (9)	0.0180 (10)	0.0230 (10)	−0.0005 (8)	−0.0024 (8)	0.0012 (8)
P1	0.0149 (2)	0.0125 (2)	0.0184 (2)	−0.0002 (2)	−0.00181 (17)	−0.00174 (19)
O2B	0.0221 (7)	0.0141 (7)	0.0247 (7)	−0.0016 (5)	−0.0009 (6)	−0.0013 (6)
N2B	0.0176 (8)	0.0166 (8)	0.0211 (8)	0.0007 (6)	−0.0062 (7)	0.0024 (6)
N4B	0.0166 (8)	0.0137 (8)	0.0174 (8)	0.0017 (6)	−0.0024 (6)	0.0021 (6)
N3B	0.0182 (8)	0.0160 (8)	0.0216 (8)	0.0006 (7)	−0.0019 (7)	0.0008 (6)
C2B	0.0171 (9)	0.0157 (9)	0.0169 (9)	0.0012 (7)	0.0027 (8)	0.0018 (7)
C3B	0.0124 (8)	0.0189 (9)	0.0155 (9)	−0.0011 (7)	0.0007 (7)	0.0011 (7)
O3	0.0214 (7)	0.0151 (6)	0.0197 (7)	−0.0021 (5)	−0.0041 (6)	0.0010 (5)
C3A	0.0188 (9)	0.0161 (10)	0.0199 (10)	0.0015 (7)	0.0014 (8)	−0.0001 (7)
O2	0.0193 (7)	0.0136 (6)	0.0412 (9)	0.0006 (5)	0.0063 (6)	0.0013 (6)
O1	0.0266 (8)	0.0177 (8)	0.0519 (10)	0.0006 (6)	−0.0217 (8)	−0.0017 (7)
N3A	0.0194 (8)	0.0145 (8)	0.0215 (8)	0.0002 (6)	0.0006 (7)	−0.0010 (6)
N2A	0.0226 (9)	0.0121 (7)	0.0239 (8)	0.0019 (6)	−0.0006 (7)	0.0013 (6)
O2A	0.0202 (7)	0.0217 (7)	0.0249 (7)	−0.0009 (6)	−0.0033 (6)	−0.0024 (6)
N1A	0.0279 (10)	0.0158 (9)	0.0311 (10)	0.0014 (7)	−0.0072 (8)	0.0009 (7)
C1B	0.0161 (9)	0.0146 (9)	0.0191 (9)	0.0012 (7)	0.0047 (7)	−0.0001 (7)
N4A	0.0184 (9)	0.0107 (8)	0.0232 (8)	0.0004 (6)	−0.0009 (7)	−0.0019 (6)
O1B	0.0181 (7)	0.0147 (6)	0.0194 (7)	−0.0009 (5)	−0.0042 (5)	0.0000 (5)
O4	0.0594 (12)	0.0425 (11)	0.0327 (9)	−0.0305 (9)	0.0219 (9)	−0.0148 (7)
O1A	0.0214 (7)	0.0154 (7)	0.0358 (8)	0.0041 (6)	−0.0132 (7)	−0.0001 (6)
C1A	0.0134 (9)	0.0166 (9)	0.0228 (10)	−0.0001 (7)	0.0003 (8)	−0.0010 (7)
C2A	0.0149 (9)	0.0168 (9)	0.0201 (9)	−0.0003 (7)	0.0020 (7)	−0.0022 (7)

### Geometric parameters (Å, º)

**Table:**

O6—N1	1.247 (2)	N2B—N3B	1.358 (2)
N3C—C2C	1.292 (3)	N2B—H2B	0.86
N3C—N2C	1.368 (2)	N4B—C3B	1.342 (3)
O1C—C1C	1.245 (3)	N4B—C2B	1.362 (3)
N1B—C3B	1.320 (3)	N4B—H4B	0.86
N1B—H5B	0.86	N3B—C2B	1.303 (3)
N1B—H3B	0.86	C2B—C1B	1.482 (3)
N4C—C3C	1.333 (3)	C3A—N1A	1.314 (3)
N4C—C2C	1.365 (3)	C3A—N2A	1.339 (3)
N4C—H4C	0.86	C3A—N4A	1.343 (2)
N1—O7	1.241 (2)	O2—H2	0.82
N1—O5	1.248 (2)	N3A—C2A	1.292 (3)
C3C—N1C	1.311 (3)	N3A—N2A	1.367 (2)
C3C—N2C	1.337 (3)	N2A—H2A	0.86
O2C—C1C	1.238 (3)	O2A—C1A	1.222 (2)
N2C—H2C	0.86	N1A—H5A	0.86
C1C—C2C	1.493 (3)	N1A—H3A	0.86
N1C—H5C	0.86	C1B—O1B	1.290 (2)
N1C—H3C	0.86	N4A—C2A	1.372 (3)
P1—O1	1.5068 (16)	N4A—H4A	0.86
P1—O3	1.5156 (14)	O1B—H1B	0.82
P1—O4	1.5371 (16)	O4—H4	0.82
P1—O2	1.5402 (14)	O1A—C1A	1.266 (2)
O2B—C1B	1.215 (2)	O1A—H1A	0.82
N2B—C3B	1.336 (3)	C1A—C2A	1.494 (3)
			
C2C—N3C—N2C	104.11 (16)	C3B—N4B—H4B	126.7
C3B—N1B—H5B	120	C2B—N4B—H4B	126.7
C3B—N1B—H3B	120	C2B—N3B—N2B	103.72 (16)
H5B—N1B—H3B	120	N3B—C2B—N4B	111.91 (17)
C3C—N4C—C2C	107.38 (17)	N3B—C2B—C1B	121.13 (17)
C3C—N4C—H4C	126.3	N4B—C2B—C1B	126.94 (17)
C2C—N4C—H4C	126.3	N1B—C3B—N2B	126.42 (18)
O7—N1—O6	120.32 (16)	N1B—C3B—N4B	127.92 (18)
O7—N1—O5	119.72 (16)	N2B—C3B—N4B	105.64 (17)
O6—N1—O5	119.94 (18)	N1A—C3A—N2A	126.59 (19)
N1C—C3C—N4C	128.80 (19)	N1A—C3A—N4A	127.73 (19)
N1C—C3C—N2C	125.70 (18)	N2A—C3A—N4A	105.69 (17)
N4C—C3C—N2C	105.49 (17)	P1—O2—H2	109.5
C3C—N2C—N3C	111.56 (16)	C2A—N3A—N2A	104.42 (16)
C3C—N2C—H2C	124.2	C3A—N2A—N3A	111.50 (16)
N3C—N2C—H2C	124.2	C3A—N2A—H2A	124.3
O2C—C1C—O1C	127.77 (19)	N3A—N2A—H2A	124.3
O2C—C1C—C2C	115.2 (2)	C3A—N1A—H5A	120
O1C—C1C—C2C	117.04 (18)	C3A—N1A—H3A	120
C3C—N1C—H5C	120	H5A—N1A—H3A	120
C3C—N1C—H3C	120	O2B—C1B—O1B	127.51 (19)
H5C—N1C—H3C	120	O2B—C1B—C2B	119.06 (18)
N3C—C2C—N4C	111.43 (17)	O1B—C1B—C2B	113.41 (16)
N3C—C2C—C1C	122.84 (19)	C3A—N4A—C2A	106.82 (16)
N4C—C2C—C1C	125.72 (19)	C3A—N4A—H4A	126.6
O1—P1—O3	113.02 (9)	C2A—N4A—H4A	126.6
O1—P1—O4	107.73 (11)	C1B—O1B—H1B	109.5
O3—P1—O4	111.50 (8)	P1—O4—H4	109.5
O1—P1—O2	108.65 (9)	C1A—O1A—H1A	109.5
O3—P1—O2	107.95 (8)	O2A—C1A—O1A	129.23 (19)
O4—P1—O2	107.83 (10)	O2A—C1A—C2A	116.94 (17)
C3B—N2B—N3B	112.03 (16)	O1A—C1A—C2A	113.83 (17)
C3B—N2B—H2B	124	N3A—C2A—N4A	111.56 (18)
N3B—N2B—H2B	124	N3A—C2A—C1A	123.02 (18)
C3B—N4B—C2B	106.68 (16)	N4A—C2A—C1A	125.42 (17)
			
C2C—N4C—C3C—N1C	−178.8 (2)	C2B—N4B—C3B—N1B	−179.47 (19)
C2C—N4C—C3C—N2C	1.5 (2)	C2B—N4B—C3B—N2B	−0.86 (19)
N1C—C3C—N2C—N3C	179.1 (2)	N1A—C3A—N2A—N3A	178.89 (19)
N4C—C3C—N2C—N3C	−1.2 (2)	N4A—C3A—N2A—N3A	−0.6 (2)
C2C—N3C—N2C—C3C	0.4 (2)	C2A—N3A—N2A—C3A	−0.1 (2)
N2C—N3C—C2C—N4C	0.6 (2)	N3B—C2B—C1B—O2B	7.5 (3)
N2C—N3C—C2C—C1C	−178.60 (19)	N4B—C2B—C1B—O2B	−174.60 (18)
C3C—N4C—C2C—N3C	−1.3 (2)	N3B—C2B—C1B—O1B	−171.11 (17)
C3C—N4C—C2C—C1C	177.8 (2)	N4B—C2B—C1B—O1B	6.8 (3)
O2C—C1C—C2C—N3C	1.2 (3)	N1A—C3A—N4A—C2A	−178.5 (2)
O1C—C1C—C2C—N3C	−177.4 (2)	N2A—C3A—N4A—C2A	1.0 (2)
O2C—C1C—C2C—N4C	−177.9 (2)	N2A—N3A—C2A—N4A	0.8 (2)
O1C—C1C—C2C—N4C	3.5 (3)	N2A—N3A—C2A—C1A	−178.54 (16)
C3B—N2B—N3B—C2B	−0.7 (2)	C3A—N4A—C2A—N3A	−1.1 (2)
N2B—N3B—C2B—N4B	0.1 (2)	C3A—N4A—C2A—C1A	178.15 (18)
N2B—N3B—C2B—C1B	178.28 (17)	O2A—C1A—C2A—N3A	7.6 (3)
C3B—N4B—C2B—N3B	0.5 (2)	O1A—C1A—C2A—N3A	−172.49 (19)
C3B—N4B—C2B—C1B	−177.57 (19)	O2A—C1A—C2A—N4A	−171.63 (18)
N3B—N2B—C3B—N1B	179.60 (19)	O1A—C1A—C2A—N4A	8.3 (3)
N3B—N2B—C3B—N4B	1.0 (2)		

### Hydrogen-bond geometry (Å, º)

**Table:**

*D*—H···*A*	*D*—H	H···*A*	*D*···*A*	*D*—H···*A*
N1*B*—H5*B*···O2*C*^i^	0.86	2.15	2.826 (2)	135
N1*B*—H5*B*···O1^ii^	0.86	2.35	2.977 (2)	130
N1*B*—H3*B*···O7	0.86	2.24	3.030 (2)	152
N1*B*—H3*B*···O1*A*	0.86	2.54	3.161 (2)	129
N4*C*—H4*C*···O5	0.86	1.92	2.765 (2)	169
N4*C*—H4*C*···O6	0.86	2.5	3.142 (2)	132
N4*C*—H4*C*···N1	0.86	2.55	3.369 (2)	160
N2*C*—H2*C*···O2*A*^iii^	0.86	2.21	2.876 (2)	135
N2*C*—H2*C*···N3*A*^iii^	0.86	2.22	2.971 (2)	146
N1*C*—H5*C*···O6	0.86	2.28	3.035 (2)	146
N1*C*—H5*C*···O1*B*	0.86	2.5	3.081 (2)	126
N1*C*—H3*C*···O2*A*^iii^	0.86	2.17	2.888 (2)	140
N1*C*—H3*C*···O3	0.86	2.61	3.224 (2)	129
N2*B*—H2*B*···O2*C*^i^	0.86	2.03	2.706 (2)	135
N2*B*—H2*B*···N3*C*^i^	0.86	2.27	3.004 (2)	144
N4*B*—H4*B*···O6	0.86	1.92	2.780 (2)	177
N4*B*—H4*B*···O7	0.86	2.66	3.276 (2)	130
N4*B*—H4*B*···N1	0.86	2.64	3.444 (2)	157
O2—H2···O1*C*^iv^	0.82	1.77	2.5563 (19)	160
N2*A*—H2*A*···O2*B*^v^	0.86	2.17	2.858 (2)	137
N2*A*—H2*A*···N3*B*^v^	0.86	2.32	3.071 (2)	146
N1*A*—H5*A*···O2*B*^v^	0.86	2.2	2.918 (2)	140
N1*A*—H5*A*···O2^v^	0.86	2.6	3.244 (2)	133
N1*A*—H3*A*···O5	0.86	2.26	3.022 (2)	148
N1*A*—H3*A*···O1*C*	0.86	2.38	2.987 (2)	129
N4*A*—H4*A*···O7	0.86	1.93	2.784 (2)	172
N4*A*—H4*A*···O5	0.86	2.52	3.157 (2)	132
N4*A*—H4*A*···N1	0.86	2.57	3.388 (2)	160
O1*B*—H1*B*···O3	0.82	1.65	2.4648 (19)	175
O4—H4···O3^vi^	0.82	1.92	2.671 (2)	151
O1*A*—H1*A*···O1^ii^	0.82	1.62	2.423 (2)	166

Symmetry codes: (i) *x*+1/2, *y*−1/2, *z*+1; (ii) *x*+1/2, *y*+1/2, *z*+1; (iii) *x*−1/2, *y*−1/2, *z*−1; (iv) *x*, *y*−1, *z*; (v) *x*, *y*+1, *z*; (vi) *x*, −*y*+2, *z*+1/2.
